# GPU-Accelerated GLRLM Algorithm for Feature Extraction of MRI

**DOI:** 10.1038/s41598-019-46622-w

**Published:** 2019-07-26

**Authors:** Hanyu Zhang, Che-Lun Hung, Geyong Min, Jhih-Peng Guo, Meiyuan Liu, Xiaoye Hu

**Affiliations:** 1 Affiliated Cancer Hospital & Institute of Guangzhou Medical University, Guangzhou, China; 2 College of Computing and Informatics, Providence University, Taichung, Taiwan; 3 Department and Graduate Institute of Computer Science and Information Engineering, Chang Gung University, Tao-Yuan City, Taiwan; 4 Division of Rheumatology, Allergy and Immunology, Chang Gung Memorial Hospital, Taoyuan City, Taiwan; 5 AI Innovation Research Center, Chang Gung University, Taoyuan City, Taiwan; 6 Department of Computer Science and Communication Engineering Providence University, Taichung, Taiwan; 7 Laboratoire Mathématiques et Informatique pour la Complexité et les Systèmes, CentraleSupélec, 91190 Gif-sur-Yvette, France; 8 Department of Mathematics and Computer Science, University of Exeter, North Park Road, Exeter, EX4 4QF UK

**Keywords:** Computational science, Computational science, Computer science, Computer science

## Abstract

The gray level run length matrix (GLRLM) whose entries are statistics recording distribution and relationship of images pixels is a widely used method for extracting statistical features for medical images, e.g., magnetic resonance (MR) images. Recently these features are usually employed in some artificial neural networks to identify and distinguish texture patterns. But GLRLM construction and features extraction are tedious and computationally intensive while the images are too big with high resolution, or there are too many small or intermediate Regions of Interest (ROI) to process in a single image, which makes the preprocess a time consuming stage. Hence, it is of great importance to accelerate the procedure which is nowadays possible with the rapid development of massively parallel Graphics Processing Unit, i.e. the GPU computing technology. In this article, we propose a new paradigm based on mature parallel primitives for generating GLRLMs and extracting multiple features for many ROIs simultaneously in a single image. Experiments show that such a paradigm is easy to implement and offers an acceleration over 5 fold increase in speed than an optimized serial counterpart.

## Introduction

The run length method is a statistical texture analysis approach first introduced by Galloway^1^ in 1975. A large set of features based on the Gray Level Run Length Matrix (GLRLM) by this approach has been designed since then for variant applications, including classification of a set of objects^1–3^, image segmentation^4^, content based image retrieval^5^ and even for recovering the three-dimensional relations form the surface of a scene^6^, etc. Though at its early stage, the run length method has not been widely spread as they seemed to be less efficient^7,8^ than co-occurrence features^9^, Tang^10^ eventually demonstrated that the run-length matrices possess as much discriminatory information as those successful conventional texture features. Recently, statistical texture features are gaining more and more interests in analyzing medical images such as segmentation, lesion detection, etc., as human tissues are random, non-homogeneous structures with no apparent regularity, which may be best characterized in statistics^11^, therefore, they normally provide higher discrimination indexes^12^. Furthermore, the power of statistical texture features get promoted when combining with the technology of artificial neural networks^13^. In particular, GLRLM based features are reported more robust^14^, and the artificial neural networks generalize better to unseen data with GLRLM features than with others^15^. However, statistics are often computationally intensive to compute, GLRLM construction makes no differences. The situation gets even worse when an appropriate size of window must be glided pixel by pixel to scan an entire medical image, especially for small and distributive lesions. Such a huge computation poses a big problem for real-time system. For other tasks, this preprocess of GLRLM construction and feature extraction would cause the whole process time consuming. Though for a single image, it may not take much time, but for hundreds of slices from an MRI examination, it may take hours to process them. Hence, it is important and beneficial if we can accelerate it. With the rapid development of massively parallel Graphics Processing Unit (GPU) computing technology, typically the CUDA framework, there is an affordable solution by parallelizing the GLRLM construction and features extraction. Such acceleration examples are reported for Gray Level Co-occurrence Matrix (GLCM) based features. Gipp *et al*.^16^ achieved 19 fold speedup for computing GLCM and Haralick features on microscope images of 1344 × 1024 pixels and 12 bit gray level depth. Dixon and Ding^17^ reported 7 fold speedup for GLCM construction and 9.83 fold speedup for feature extraction on diffraction images. Instead of employing CUDA as mentioned in two previous literatures, Doycheva *et al*.^18^ use OpenCL with GPU to compute GLCM and to extract features for pavement distress detection, a maximum of 39 fold and 126 fold speedup are obtained, respectively. Tsai *et al*.^19^ designed highly tailored GPU computational kernels for small ROIs and pushed the speedup for GLCM generation and feature extraction together to as high as more than 200 fold for single precision and 160 fold for double precision in best scenarios on a single GPU. In these works, different techniques are employed as the underlying problems are not exactly the same. For example, in the work of Tsai *et al*., GLCM matrices and corresponding features are computed for tens of thousands ROIs in a single image, and the methods suits best for small ROIs from 6 × 6 to 9 × 9 pixels in order to fully exploit the shared memory in GPU, otherwise, performance drops significantly, whereas other works deal with the image as a single ROI. However, to the best knowledge of the authors, no literature is published on transplanting or inventing the parallel techniques to the GLRLM construction and related features extraction. Therefore, in this article, we proposed a new paradigm of GPU acceleration based on mature parallel primitives for generating GLRLMs and extracting multiple features for many ROIs simultaneously in a single image. This technique not only suits the problem we are tackling on, but may also be ported to other statistical approach of features extraction based on some kind of histograms with minor modifications.

The rest of this article is organized into five sections as follows. Section materials sets the model problem, we give a detailed description of the brain Magnetic Resonance (MR) Images that will be used to evaluate our methods. For completeness, the matrix GLRLM and the most widely used 11 features based on GLRLM are reviewed in section GLRLM and feature. Then we give our new paradigm of parallelization for GLRLM constructions and features extraction in section methods along with an optimized version of serial counterpart. Section experiments and results presents a series of experiments and empirical results of comparisons. Finally in section conclusion, we draw conclusions and give some possible perspectives of improvements.

## Materials

**Figure 1:**
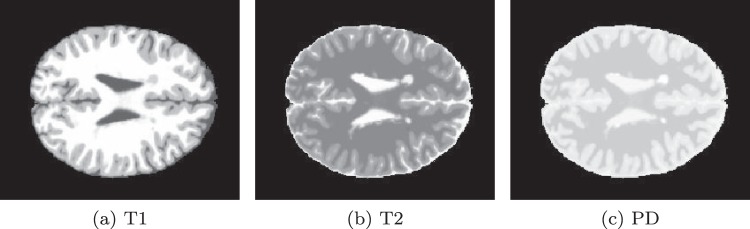
MRI brain images.

In this study, test images are chosen from the Simulated Brain Database of the Mc-Connell Brain Imaging Centre at McGill University^20^. As we are not interested in performance of classifications but merely the speedup of generation of GLRLM and features extractions, a simulated MR image would be sufficient for the goal. The database contains a set of MR brain images produced by an MRI simulator^21^. Simulation settings can be configured from 3 modalities, 5 slice thicknesses, 6 levels of noise and 3 levels of intensity non-uniformity. In general, different configurations try to simulate practical artifacts which can be used to evaluate performance of some classifier, they do not influence significantly the speed of GLRLM construction and features extractions, as for images of a fixed size, they act as the same containers of similar data, which require approximately the same amount of work, i.e., similar GLRLM construction and similar feature extractions for the same number of ROIs. Therefore, we choose to simply use the default setting of the simulator, i.e., 1 mm slice thickness, 3% noise and 20% intensity non-uniformity. The original image contains heterogeneous information, including skull part, but we removed these irrelevant information by some preprocessing, leaving only the region of brain tissue in order to stay focused. Three examples of preprocessed simulated MR brain tissue images are ready to use, shown in Fig. 1 from left to right for T1, T2, and PD pulse sequences, respectively. The images are rendered in 256 gray levels and contain 181 × 217 pixels.

## GLRLM and Features

In Gray Level Run Length Matrix (GLRLM), statistic in concern is the number of pairs of gray level value and its length of runs in a certain Region of Interest (ROI). A gray level run is a set of pixels having the same gray level value, which are consecutively and collinearly distributed in the ROI along some given directions. Number of pixels in that particular set is called the length of the gray level run. Thus a gray level value and its length of a gray level run together characterize such a set. A GLRLM is kind of a 2D histogram in form of a matrix that records the occurrence of all various combinations of gray level values and gray level runs in an ROI for a given direction. Conventionally, gray level values and gray level runs are denoted as keys of rows and columns, respectively, of the matrix, hence, the $(i,j)$-th entry in the matrix specifies the number of combinations whose gray level value is *i* and whose run length is *j*. Four principal directions are usually considered in practice, i.e., horizontal (0°), anti-diagonal (45°), vertical (90°) and diagonal (135°). For example, consider an ROI as shown in Table 1, then we compute and list its corresponding GLRLMs along 4 principal directions in Table 2, where *V* represents the gray level value and *L* stands for run length. Notice that it is sufficient to list only 5 non null rows in the GLRLMs along the four main directions, as there are only 5 different gray level values.

**Table 1:** An example of ROI.

0	255	113	113	42
255	42	113	113	0
128	113	255	0	42
113	255	0	128	42
42	113	128	255	255

**Table 2:** GLRLMs along 4 main directions of the ROI example in Table 1.

*V*\*L*	1	2	3	4	5
**(a) 0° horizontal**					
0	4	0	0	0	0
42	5	0	0	0	0
113	3	2	0	0	0
128	3	0	0	0	0
255	4	1	0	0	0
**(b) 45° anti-diagonal**					
*V*\*L*	1	2	3	4	5
0	1	0	1	0	0
42	5	0	0	0	0
113	3	0	0	1	0
128	1	1	0	0	0
255	2	2	0	0	0
**(c) 90° vertical**					
*V*\*L*	1	2	3	4	5
0	4	0	0	0	0
42	3	1	0	0	0
113	3	2	0	0	0
128	3	0	0	0	0
255	6	0	0	0	0
**(d) 135° diagonal**					
*V*\*L*	1	2	3	4	5
0	4	0	0	0	0
42	5	0	0	0	0
113	3	2	0	0	0
128	3	0	0	0	0
255	6	0	0	0	0

By convention, we use *P* to denote a GLRLM, then *P*_*ij*_ is the $(i,j)$-th entry of the GLRLM. In addition, we use *N*_*r*_ to denote the set of different run lengths that actually occur in the ROI, and *N*_*g*_ the set of different gray levels exist in the ROI. And finally let *N* be the number of total pixels in the ROI, then clearly we shall have the following equality,

1 $$N=\sum _{i\in {N}_{g}}\,\sum _{j\in {N}_{r}}\,j{P}_{i,j}$$

which can be used as a simple verification for correctness of a GLRLM. Historically many run length based features has been designed. Galloway^1^ proposed 5 features to classify the same set of terrain samples that were also studied by Haralick^9^ and obtained quite promising results. These features are listed here, we refer readers to their work^1^ for detailed explanations.

- Long Runs Emphasis
  2 $$LRE=\sum _{i\in {N}_{g}}\,\sum _{j\in {N}_{r}}\,{j}^{2}{P}_{ij}/\sum _{i\in {N}_{g}}\,\sum _{j\in {N}_{r}}\,{P}_{ij}$$
- Short Runs Emphasis
  3 $$SRE=\sum _{i\in {N}_{g}}\,\sum _{j\in {N}_{r}}\,\frac{{P}_{ij}}{{j}^{2}}/\sum _{i\in {N}_{g}}\,\sum _{j\in {N}_{r}}\,{P}_{ij}$$
- Gray Level Nonuniformaity
  4 $$GLN=\sum _{i\in {N}_{g}}\,{(\sum _{j\in {N}_{r}}{P}_{ij})}^{2}/\sum _{i\in {N}_{g}}\,\sum _{j\in {N}_{r}}\,{P}_{ij}$$
- Run Length Non-uniformity
  5 $$RLN=\sum _{j\in {N}_{r}}\,{(\sum _{i\in {N}_{g}}{P}_{ij})}^{2}/\sum _{i\in {N}_{g}}\,\sum _{j\in {N}_{r}}\,{P}_{ij}$$
- Run Percentage
  6 $$RP=\sum _{i\in {N}_{g}}\,\sum _{j\in {N}_{r}}\,{P}_{ij}/N$$
  Having observed the symmetrical roles played by gray levels *i* and run length *j*, Chu *et al*.^3^ proposed, analogically to SRE (3) and LRE (2), the following 2 features, which use the gray level distribution of runs instead and are demonstrated to be potentially valuable in classification.
- Low Gray Level Run Emphasis
  7 $$LGRE=\sum _{i\in {N}_{g}}\,\sum _{j\in {N}_{r}}\,\frac{{P}_{ij}}{{i}^{2}}/\sum _{i\in {N}_{g}}\,\sum _{j\in {N}_{r}}\,{P}_{ij}$$
- High Gray Level Run Emphasis
  8 $$HGRE=\sum _{i\in {N}_{g}}\,\sum _{j\in {N}_{r}}\,{i}^{2}{P}_{ij}/\sum _{i\in {N}_{g}}\,\sum _{j\in {N}_{r}}\,{P}_{ij}$$
  Following the invention of LGRE (7) and HGRE (8), Dasarathy and Holder^2^ caught the idea of using distributions of gray level and run length jointly to propose 4 new features as follows. Experiments show that they are more effective when comparing to the features that separately employ either gray level or run length.
- Short Run Low Gray Level Emphasis
  9 $$SRLGE=\sum _{i\in {N}_{g}}\,\sum _{j\in {N}_{r}}\,\frac{{P}_{ij}}{{i}^{2}{j}^{2}}/\sum _{i\in {N}_{g}}\,\sum _{j\in {N}_{r}}\,{P}_{ij}$$
- Short Run High Gray Level Emphasis
  10 $$SRHGE=\sum _{i\in {N}_{g}}\,\sum _{j\in {N}_{r}}\,\frac{{i}^{2}{P}_{ij}}{{j}^{2}}/\sum _{i\in {N}_{g}}\,\sum _{j\in {N}_{r}}\,{P}_{ij}$$
- Long Run Low Gray Level Emphasis
  11 $$LRLGE=\sum _{i\in {N}_{g}}\,\sum _{j\in {N}_{r}}\,\frac{{j}^{2}{P}_{ij}}{{i}^{2}}/\sum _{i\in {N}_{g}}\,\sum _{j\in {N}_{r}}\,{P}_{ij}$$
- Long Run High Gray Level Emphasis

12 $$LRHGE=\sum _{i\in {N}_{g}}\,\sum _{j\in {N}_{r}}\,{i}^{2}{j}^{2}{P}_{ij}/\sum _{i\in {N}_{g}}\,\sum _{j\in {N}_{r}}\,{P}_{ij}$$

There is no doubt that all of the features defined above belong to the same category according to their appearance and their historical development. Hence in this article, we are interested in extracting these 11 features listed above in a uniform way.

## Methods

In this section, we present our methods in two parts. First we establish an optimized workflow of sequential counterpart to lay the benchmark for comparisons. Then we discuss how to transform the problems of GLRLM construction and features extraction into a form that is possible to leverage the computational power of GPU based on mature parallel primitives for acceleration.

### Sequential counterpart

We break the sequential program into two parts. One for the construction of GLRLM and the other for features extraction. We will define some terms in this section to make the text more precise and easy to read, which will also be used in discussing parallel accelerations.

Notice that we actually compute GLRLM and features in ROIs, we should distinguish its width and height from those of the entire image. Therefore we denote ROI’s width by roi_width, but simply with width for the entire image’s width which are measured by column numbers. Similarly, roi_height and height are used for ROI’s height and image’s height, respectively, which are measured by row numbers. A pair in form (dx, dy) is used to denote the direction. By following the convention in image processing that the $(0,0)$ pixel is the left-up corner, and the positive *x* axis points to the right while the positive *y* axis points downside, we may deduce that $(dx,dy)=(1,0)$ means horizontal (0°) direction and $(1,-\,1)$, $(0,-\,1)$, $(\,-\,1,-\,1)$ represent the directions of anti-diagonal (45°), vertical (90°) and diagonal (135°), respectively. By this way, a consecutive neighbor pixel to a given pixel $(x,y)$ along certain direction $(dx,dy)$ can be easily calculated by $(x+dx,y+dy)$.

Furthermore, a gray level value and run length pair may not be always stored efficiently in memory as we need two aligned arrays, we use a one-to-one mapping to map the pair into an integer as follows,

$${\rm{index}}=\mathrm{gray}\_\mathrm{level}\times \mathrm{roi}\_\mathrm{length}+\mathrm{run}\_\mathrm{length}-{\rm{1}}$$

where roi_length is the maximum possible run length of a ROI along a given direction. And it is also convenient to restore the gray level or the run length by a single integer division or module operation respectively. Now we are prepared to give the optimized sequential algorithm for GLRLM constructions and features extractions.

**Algorithm 1:**
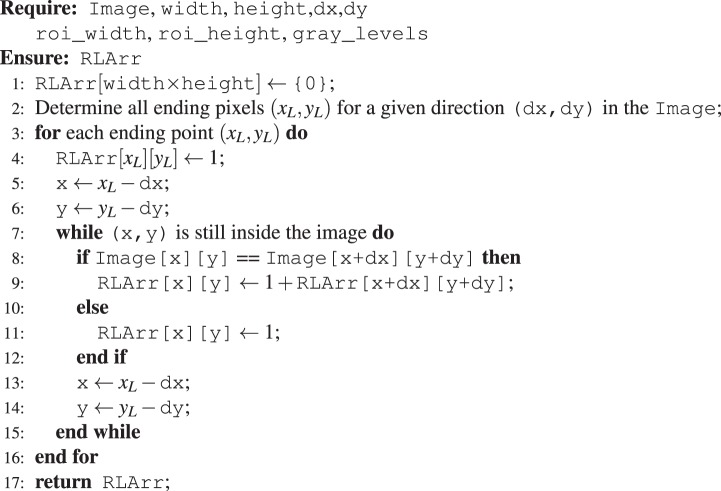
Counting run length for each pixel.

#### Sequential GLRLM construction

**Figure 2:**
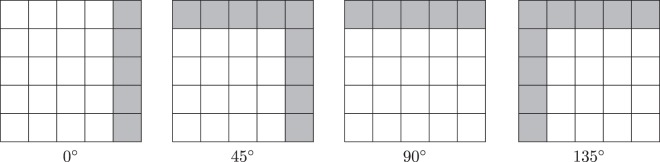
Illustration of ending pixels for different directions.

The GLRLM constructions are split into two steps. As ROIs are glided pixel by pixel to scan over the entire image, they are heavily overlapped, so is the gray level values and run length pairs. If we construct GLRLM for each ROI one by one by following each pixel inside the ROI to count run lengths, there will be a lot of repeating works, which are redundant. Therefore we propose to count run length only once along a given direction for the entire image and store that information in a separate array which is of the same shape as the image, we shall refer to it as the run length array or RLArr. The main idea is that for each pixel in the image, taking that pixel as the starting point along the given direction (dx, dy), we count its run length and then we store the run length value into the corresponding entry in the run length array. This process can be done with linear complexity to the size of image and constant extra memory besides the run length array, see Algorithm 1. Notice that we must iterate through each pixel reversely to the given direction, thus we first find out the ending pixels in the entire image for a given direction. See the illustration of ending pixels in Fig. 2. It is quite easy to determine the ending pixels according to direction and image shape. Take (dx, dy) = (1, −1) for example, then all ending pixels are

$$\begin{array}{rcl}\{({x}_{L},{y}_{L}):{x}_{L} & = & {\mathtt{width}}-1,{y}_{L}\in [[0,{\mathtt{height}}-1]]\}\\  &  & \cup \{({x}_{L},{y}_{L}):{x}_{L}\in [[0,{\mathtt{width}}-2]],{y}_{L}=0\}\end{array}$$

Once ending pixels are resolved, the rest part of Algorithm 1 is easy to follow. Having computed the run length array, whenever we need the run length for a pixel, starting from it along a given direction in a ROI, it is sufficient to read the corresponding entry in the run length array and compare it to the distance (in pixels) to the boundary of the ROI, then take the smaller one. Furthermore, we can skip the following consecutive pixels of the same gray level value directly to the next starting pixel of another gray level value, which saves a considerable amount of time comparing to a naive implementation.

#### Sequential feature extraction

After constructing the GLRLM, features extraction is straight forward according to equations (2)–(12), notice that all the features share the same denominator, thus it is calculated only once and passed as an argument to compute the features.

### Parallel acceleration

With the rapid development of the GPU computing technology, it has been much easier to obtain acceleration from parallel computing now than the very beginning of this parallel tendency. Especially CUDA (Compute Unified Device Architecture), which is both a parallel computing platform and application programming interface (API) model, created by Nvidia, allows developers and engineers to use GPU for general purpose programming. Based on CUDA, parallel primitives that are widely studied and carefully designed for particular parallel pattens have been implemented and integrated in various libraries. In this paper we explorer the possibility of employing only parallel primitives to accomplish the complicated tasks like GLRLM constructions and features extraction of multiple ROIs for a single image, and investigate the acceleration gained by the approach. We shall first review briefly the GPU architecture and CUDA model, then introduce the CUB library, mainly with which we accomplished the task.

#### GPU and CUDA

GPU differs from CPU mainly in two aspects. One for the multiple stream multiprocessors (SMs) possessed by GPU, which usually contains hundreds of integrated simple cores, each can run a thread in parallel, hence a GPU with dozens of SMs can easily launch thousands of threads simultaneously. Another one is the manageable hierarchy of memory organization at developer’s disposal, which means that explicit designation of usage can be made for registers, separated shared memory, constant cache, texture cache and L1/L2 cache, local memory for each SM and global memory for entire device of very large bandwidth. Therefore, it’s up to developers to assign particular data patterns to appropriate memory. Nvidia’s CUDA platform, which may be the earliest widely adopted software layer that gives direct access to the GPU’s virtual instruction set and parallel computational elements, makes the general purpose programming on GPU relatively easy. Generally speaking, CUDA enables custom functions to be executed in parallel on GPU’s SMs. These custom functions are called compute kernels, which can be directly defined in a normal C program as CUDA provide an extensions to the C programming language. Hence parallel primitives are generally implemented in this way and can be called as a function. Nowadays there are abundant literatures^22–24^ talking about how to program GPU with CUDA in great details, therefore, we suggest readers to refer to them.

#### CUB library

Though CUDA provides the tool set to write general purpose program to accomplish complicated tasks, it doesn’t mean that one can easily implement an efficient program to solve a particular problem, as parallel modeling is quite different from the sequential counterpart and flexible management of memory also makes it difficult to be handled appropriately. Hence there exists various libraries based on CUDA that implements common collective primitives that take care of low-level details and leave user to deal with main logic of problems. Example primitives like parallel sort, prefix scan, reduction, histogram, etc. are essential for constructing high-performance, maintainable parallel programs.

**Figure 3:**
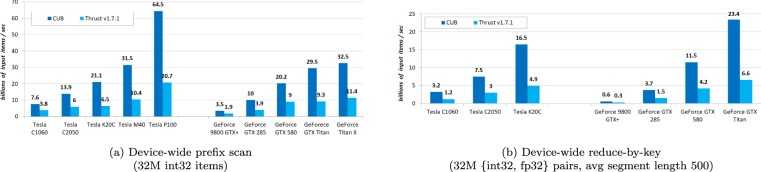
Performance-portability comparisons (reproduced according to CUB’s official website^25^) between libraries CUB and Thrust.

CUB is such a library that provides state-of-the-art, reusable software components for every layer of the CUDA programming model, including warp-wide “collective” primitives, block-wide “collective” primitives and device-wide primitives^25^. Furthermore, CUB’s collective primitives are not bound to any particular width of parallelism or data type, developers can quickly change grain size and switch to alternative algorithmic strategies to best match the processors resources for their target architecture. Finally, most of CUB’s implementation of primitives, at least for what we employed in this work, are much faster than the famous traditional, rigidly-coded parallel library Thrust in performance-portability comparisons, as reported in Fig. 3. That’s also why we choose library CUB over Thrust. Brief, we are able to speedup the implementation of prototypes and get a decent acceleration with CUB, which increase the efficiency of development.

#### Parallel GLRLM construction

**Figure 4:**
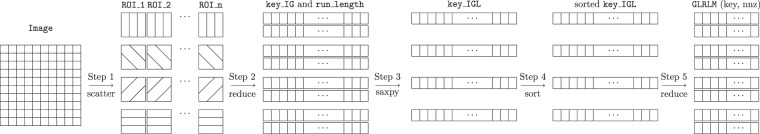
Flow chart of construction of GLRLMs for all ROIs in parallel.

In this section, we demonstrate how straight forward it can be to construct the GLRLMs simultaneously for all ROIs in a single image by repeatedly applying some collective primitives with CUB. Though such an implementation may not be optimal in performance, the main purpose is to show the possibility to program a prototype really fast and yet with a reasonable acceleration in performance. In Fig. 4, we give a flow chart of how to simultaneously construct GLRLMs for all ROIs in an image. There are in total five steps to complete the construction. First we need to copy all ROIs in the image into GPU memory, with these pixels duplicated and rearranged appropriately in the GPU memory, we are then able to use a primitive called reduce-by-key to count the gray level run length for all ROIs in the given direction. Then we combine the gray level and its run length to generate a new key. After which we sort the array of new keys to bring segments of the same ROI together so that we can use another reduce-by-key to construct all GLRLMs at the end. In the whole process, data are conceptually stored in the main memory of GPU, as we only need to care about the high-level logic to accomplish the task and leave the low-level details to the CUB library, like shared memory’s usage in the sort or splittings of blocks in the reduction. In the following paragraphs, we look into the five steps in depth with some illustrations to make things more clear.

**Figure 5:**
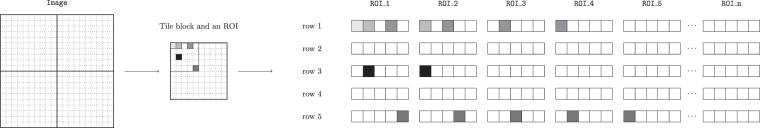
Illustration of the first step, several pixels are colored to show their movements.

**Step 1** In the first step, we separate all ROIs and spread them into the main memory of GPU. As ROIs are heavily overlapped, each pixel will be copied for as many times as the number of ROIs who contains it. This is the only place where parallel primitives can not help, thus we first copy tile blocks in the image into the shared memory to avoid multiple accesses to main memory. The shape of tile block is calculated dynamically according to the number of SMs and the size of shared memory, so that available resources are utilized as much as possible. Then we scatter the tile blocks to the right places, which is illustrated in Fig. 5. In this illustration, the case of horizontal direction (0°) is presented, but we use the term “row” to mean consecutive pixels along a given direction, so that in the following explanation, the idea is general and it can easily be adapted to other cases. There are two principles that must be satisfied in scattering so that crucial information is not lost or altered. First, each row in an ROI must be kept consecutive for counting run length. Second, different rows in an ROI should be separated in case that the ending pixel of one row has the same gray level value as the starting pixel of the next row. Therefore, we rearrange the pixels in such a way that rows of all ROIs are concatenated, all first rows first, then all second rows, third rows, etc. Let us denote an ROI by its index, which can be calculated from its top-left pixel’s row and column indices $(i,j)$ as ROI_index = *i* × width + *j*, then for the $(l+1)$-th entry in the $(k+1)$-th row in an ROI, it should be scattered to the place,

13 $${N}_{k}\times \mathrm{num}{\mathtt{\_}}\mathrm{ROIs}+{R}_{k}\times \mathrm{ROI}{\mathtt{\_}}\mathrm{index}+l$$

where num_ROIs is the total number of ROIs in the input image, *R*_*k*_ is the number of entries for the $(k+1)$-th row and ${N}_{k}={\sum }_{i=0}^{k-1}\,{R}_{k}$ is the number of entries in the first *k* rows. Now for a particular pixel in the tile block, it is sufficient to figure out its position in an enclosing ROI and the ROI’s index. Remember that there are more than one ROIs containing the same pixel, thus in the meantime of scattering, the ROI’s index is attached to gray level value as follows,

14 $$\mathrm{key}{\mathtt{\_}}\mathrm{IG}:=\mathrm{ROI}{\mathtt{\_}}\mathrm{index}\oplus \mathrm{gray}{\mathtt{\_}}\mathrm{level}=\mathrm{ROI}{\mathtt{\_}}\mathrm{index}\times \mathrm{GL}{\mathtt{\_}}\mathrm{RESOLUTION}+\mathrm{gray}{\mathtt{\_}}\mathrm{level}$$

where GL_RESOLUTION is the number of gray levels, which is 256 in our case. And gray_level is the gray level value of a particular pixel. We then use key_IG and symbol ⊕ as the indicator and operation to denote the paired information of ROI indices and gray level values. In this way, important information are retained, and we are now ready to use parallel primitives.

**Figure 6:**
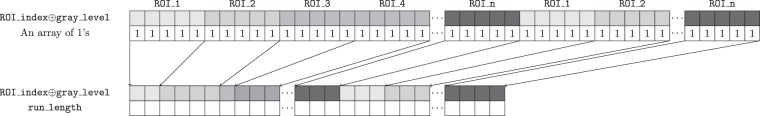
Illustration of the second step, a reduce-by-key leaves a single key_IG for consecutive ones and count its run length once for all.

**Step 2** Once the array of key_IG is set, we can directly apply CUB’s routine reduce-by-key to count the run length of gray level values along a given direction for all ROIs at the same time. As illustrated in Fig. 6, different ROIs will have different key_IG, while the same gray level value in the same ROI will always have the same key_IG, and in addition, spatial structure in rows for gray level values is kept exactly the same as in the array of key_IG by our design. Hence, the primitive reduce-by-key for a one-array with key array of key_IG is equivalent to count the run length of consecutive gray level values in all ROIs. At the end of this step, the size of array will be greatly reduced.

**Figure 7:**
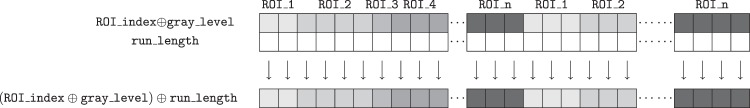
Illustration of the third step, a parallel saxpy combines key_IG and run_length as key_IGL.

**Step 3** After the first reduction, pairs of existing gray level values and its run lengths are found for each ROI. However, these pairs are spread over the entire array even for a single ROI, which makes it troublesome to count the number of unique pairs for all ROIs. Moreover, moving two arrays’ elements around means two times of memory accesses, especially when they are not coalesced, it may worth to do some extra arithmetic computations to reduce memory accesses. Therefore, we decided to use again the same strategy as in the first step to attach the key_IG to its run length as follows

15 $$\mathrm{key}{\mathtt{\_}}\mathrm{IGL}:=\mathrm{key}{\mathtt{\_}}\mathrm{IG}\oplus \mathrm{run}{\mathtt{\_}}\mathrm{length}=\mathrm{key}{\mathtt{\_}}\mathrm{IG}\times \mathrm{roi}{\mathtt{\_}}\mathrm{length}+\mathrm{run}{\mathtt{\_}}\mathrm{length}-{\mathtt{1}}$$

where roi_length is larger value between roi_width and roi_height. As a result, all information of ROI’s index, pairs of gray level value and run length are now integrated into a single integer. As shown in Fig. 7, this new array of key_IGL actually stores every occurrence of gray level value and run length pairs for all ROIs.

**Step 4** Now we need to count the number of different key_IGL values, which is in fact the same task as computing the non nulls entries in the GLRLMs. However, we must collect key_IGL for each ROI first. One possible way is to sort the array of key_IGL, as ROI’s index are also embedded inside as the most significant bits in the key.

**Figure 8:**
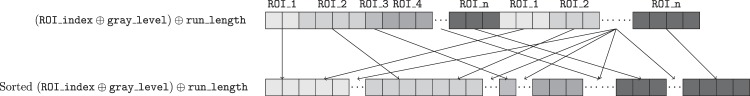
Illustration of the forth step, key_IGL are sorted so that keys are clustered for each ROI.

This step is illustrated in Fig. 8. We should also remark that not only the same ROI’s key_IGL are gathered together, but the same pairs of gray level value and run length are also clustered, which can be observed from the figure as well.

**Figure 9:**
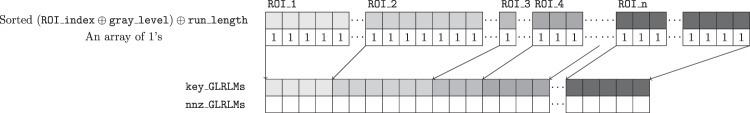
Illustration of the fifth step, all GLRLMs are generated in two arrays with only non null entries.

**Step 5** Finally we can apply again the CUB’s primitive of reduce-by-key on the sorted array of key_IGL with a one-array. As shown in Fig. 9, the resulting two arrays are exactly the GLRLMs for all ROIs that we are looking for, naturally containing only non null entries. We would refer to this reduced array of key_IGL as key_GLRLMs and the array of non null entries as nnz_GLRLMs.

#### Parallel feature extraction

Now we can turn to the stage of extracting 11 features from the generated GLRLMs. The general idea is to transform arrays of keys_GLRLMs and nnz_GLRLMs to get new arrays of key-value pair so that we can apply a reduce-by-key to calculate the sums in features for all ROIs in parallel. We divide summations in features into three categories, as sums in the same category can be treated in the same way. The first one only contains one summation, which is the numerator of *RP* and the denominator in all features except *RP*; the second one are numerators in features of *LRE*, *SRE*, *LGRE*, *HGRE*, *SRLGE*, *SRHGE*, *LRLGE* and *LRHGE*. and the last one are numerators in features of *GLN* and *RLN*. The unique summation in first category ${\sum }_{i\in {N}_{g}}\,{\sum }_{j\in {N}_{r}}\,{P}_{ij}$ is relatively simple to calculate. It is sufficient to extract an array of ROIs’ indices from the key_GLRLMs as follows,

16 $$\mathrm{ROI}{\mathtt{\_}}\mathrm{index}=(\mathrm{key}{\mathtt{\_}}\mathrm{GLRLMs}/\mathrm{roi}{\mathtt{\_}}\mathrm{length})/\mathrm{GL}{\mathtt{\_}}\mathrm{RESOLUTION}$$

and this array of ROIs’ indices is kept for future reuse. Then another reduce-by-key on the array of ROI’s keys and nnz_GLRLMs would do the job. For the second category of summations, it is necessary to construct an array of the inner term in the feature’s summation (2),(3),(7)–(12) before applying again another reduce-by-key, which can be done by transforming nnz_GLRLMs with gray_level and/or run_length. Thus we also extract them from key_GLRLMs as

17 $$\begin{array}{rcl}\mathrm{gray}{\mathtt{\_}}\mathrm{level} & = & (\mathrm{key}{\mathtt{\_}}\mathrm{GLRLMs}/\mathrm{roi}{\mathtt{\_}}\mathrm{length}){\mathtt{ \% }}\mathrm{GL}{\mathtt{\_}}\mathrm{RESOLUTION}\\ \mathrm{run}{\mathtt{\_}}\mathrm{length} & = & \mathrm{key}{\mathtt{\_}}\mathrm{GLRLMs}{\mathtt{ \% }}\mathrm{roi}{\mathtt{\_}}\mathrm{length}+{\mathtt{1}}\end{array}$$

For the last category of summations, they are a little more complicated to compute as one must calculate a square of summation first. Hence for the inner sum in (4)-(5), we need a new array of keys that gray_level or run_length must be dropped from the key_GLRLMs for *RLN* and *GLN* respectively. Notice that dropping run_length in key_GLRLMs would still keep keys in order, thus one can directly apply a reduce-by-key, to compute the inner summation, followed by a parallel square transformation. However, dropping gray_level intermingles the order of keys, therefore another segmented sort of keys is needed to restore the clustering of keys, in the meanwhile, occurrence number should also be moved correspondingly. Then apparently a reduce-by-key, a parallel square, and another reduce-by-key in order would finish the job. At the end, a parallel division leads to the features.

## Experiments and Results

In this section, we shall present the configurations of experiments, the experiments themselves and the outcomes. We conduct a series of experiments for different ROI sizes on three inputs, mainly aiming at comparing the performance of the sequential and parallel programs. In each experiment, three input of MRI images are fed to and processed by the program one by one. But ROI sizes are considered in separate experiments. We carefully examine in each experiment the extracted features from both sequential and parallel program, and validate the equivalence between them. Then we record the time spent on different parts of computations or preparation, like GLRLM construction and feature extractions in four directions, as well as memory allocations for total execution time. All experiments are repeated for 15 times, average timing and speedups with standard deviations are reported in the following sections.

### Environments

Our experiments were conducted with two configurations of different GPUs, so that the results is not specific to a particular GPU and reveals how our parallel program adapts to different devices. Here we list some main specifics of hardware in Table 3. For the aspect of operating system and software, we use Ubuntu 16.04.4-x86_64 with kernel version 4.13.0-37-generic, and the sequential program is compiled by a GNU C-complier of version 5.4.0 with optimization flag -O3, while the parallel counterpart is compiled with CUDA-7.5 toolkits.

**Table 3:** Machine configurations.

Devices	Xeon E5-2620	TITAN V	Tesla P100
Cores/SMs	8	80	56
Threads per core/Cores per SM	2	64	64
Total threads/cores	16	5120	3584
Clock speeds (MHz)	2100	1200	1190
Main/Global memory (GB)	12	12	12
Memory bandwidth (GB/s)	19.2	652.8	732
L1 cache/Share memory (KB)	64	96	24

### Results

We first show the features obtained from the MRI images, then we report the results of performance comparisons between sequential and parallel programs in this section.

#### Feature images

**Figure 10:**
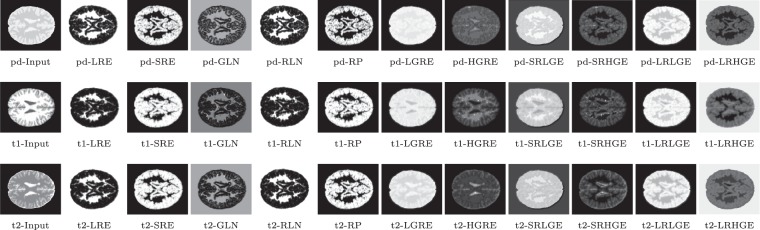
Input MRI images of three pulse sequences (PD, T1, and T2) and 11 features extracted for each.

Extracted features are presented in form of images as in Fig. 10, which may be recognized easily. It should be mentioned that it is a little difficult to visualize the textures of features LGRE, LRHGE, LRLGE, and SRLGE, as these features of ROIs are close to either white or black, thus, we apply a logarithm transformation on them to spread the values more evenly and make the visualization of textures obvious. Though input MRI images of different pulse sequences are visually quite different, features extracted from them are similar as expected, because it is the structure of textures that matters.

#### Performance comparisons

We are first interested in the general evolution of the total time elapsed from the beginning to the point features are computed, and the total speedup calculated based on it, according to the increase of ROI sizes.

**Figure 11:**
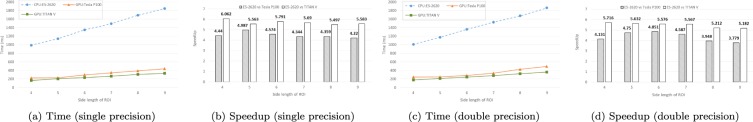
Total time and speedup evolution along ROI sizes.

Results are presented in Fig. 11 for both single precision and double precision cases. As expected, parallelization with single precision always performs better than with double precision on both devices, which is predictable since all GPUs are more efficient in single precision mode at a cost of lower precision. However, in any case, the performance of parallel program is superior to its sequential counterpart. The speedups achieved on Tesla P100 GPU are around 4.5-fold for single precision and 4.3-fold for double precision. And for device of Titan V, the speedups are around 5.7-fold and 5.5-fold for single precision and double precision, respectively. As ROI size increases, both sequential and parallel programs spend almost linearly increasing time to complete an entire task, as a result, though the speedup drops, yet it only drops asymptotically to about 4-fold.

Then we look into some details of the time comparisons. We take the typical case where ROI size equals to 4 for example. The total time is split into times of processing MRI images of pulse sequences PD, T1, and T2, as shown in Table 4 for both single and double precision. Average time needed to process different inputs are almost the same, except for the GPU Tesla P100 when dealing with MRI image of PD pulse sequence, which may be an outlier due to some unexpected coincidence within the device. And we should also mention that the performance of Titan V is more stable than that of Tesla P100.

**Table 4:** Time and speedup for 4 × 4 ROIs.

Configurations	P100	vs	Xeon E5-2620	TITAN V	vs	Xeon E5-2620	units
Input MRI image	T1	T2	PD	T1	T2	PD	
CPU time (single)	983.6_±2.3_	984.0_±5.3_	1001.6_±31.9_	983.6_±2.3_	984.0_±5.3_	1001.6_±31.9_	ms
GPU time (single)	261.4_±47.3_	250.1_±37.1_	157.1_±2.5_	171.5_±8.9_	167.9_±9.1_	150.4_±8.2_	ms
Speedup (single)	3.8_±0.7_	3.9_±0.7_	6.4_±0.2_	5.8_±0.3_	5.9_±0.3_	6.7_±0.3_	x
CPU time (double)	1005.3_±17.8_	1003.7_±26.6_	1009.2_±28.1_	1005.3_±17.8_	1003.7_±26.6_	1009.2_±28.1_	ms
GPU time (double)	270.4_±43.8_	283.1_±33.8_	177.2_±1.7_	182.5_±4.3_	168.3_±9.2_	177.2_±6.6_	ms
Speedup (double)	3.7_±0.7_	3.6_±0.4_	5.7_±0.2_	5.5_±0.2_	5.9_±0.5_	5.7_±0.2_	x

Furthermore, we also provide the individual computation time for GLRLMs construction and features extraction in 4 directions since that’s where the acceleration actually takes place. Details are shown in Table 5 for single precision and in Table 6 for double precision. Note that time for setting the device, moving data around or allocating memory, etc., is not considered in these tables, but these extra costs can be deduced by subtracting computation time from the total time recorded in Table 4.

**Table 5:** Timing and speedup for 4 × 4 ROIs with single precision.

Configurations	P100	vs	Xeon E5-2620	TITAN V	vs	Xeon E5-2620	units
	T1	T2	PD	T1	T2	PD	
CPU time	70.7_±0.5_	70.3_±0.4_	71.9_±2.3_	70.7_±0.5_	70.3_±0.4_	71.9_±2.3_	ms
GPU time	56.4_±18.3_	50.7_±11.5_	38.2_±0.5_	37.1_±2.0_	36.9_±2.7_	37.6_±2.9_	ms
GLRLM Speedup	1.3_±0.4_	1.4_±0.3_	1.9_±0.1_	1.9_±0.1_	1.5_±0.3_	1.9_±0.2_	x
CPU time	209.1_±0.4_	209.3_±1.2_	212.9_±6.9_	209.1_±0.4_	209.3_±1.2_	212.9_±6.9_	ms
GPU time	96.3_±24.1_	96.4_±27.2_	60.1_±0.9_	57.4_±3.6_	54.1_±2.6_	52.1_±4.2_	ms
0° feature Speedup	2.2_±0.6_	2.2_±0.7_	3.6_±0.1_	3.7_±0.2_	2.4_±0.7_	4.1_±0.3_	x
CPU time	209.6_±0.5_	210.1_±1.3_	214.3_±6.9_	209.6_±0.5_	210.1_±1.3_	214.3_±6.9_	ms
GPU time	37.1_±12.6_	37.1_±12.3_	19.3_±0.4_	28.5_±2.7_	28.2_±2.6_	20.8_±3.2_	ms
45° feature Speedup	5.7_±1.7_	5.7_±1.7_	11.1_±0.4_	7.4_±0.7_	7.5_±0.6_	10.5_±1.3_	x
CPU time	208.9_±0.4_	209.1_±1.2_	212.8_±6.8_	208.9_±0.4_	209.1_±1.2_	212.8_±6.8_	ms
GPU time	21.3_±7.9_	21.8_±10.3_	15.9_±0.3_	17.2_±1.5_	26.6_±1.5_	15.7_±1.5_	ms
90° feature Speedup	9.8_±2.4_	9.6_±3.3_	13.4_±0.6_	12.3_±1.1_	13.1_±1.1_	13.7_±1.3_	x
CPU time	208.5_±0.4_	209.1_±1.2_	212.4_±6.8_	208.5_±0.4_	209.1_±1.2_	212.4_±6.8_	ms
GPU time	41.2_±14.4_	34.0_±8.9_	18.7_±0.4_	25.6_±0.9_	26.6_±0.5_	18.5_±1.3_	ms
135° feature Speedup	5.1_±1.9_	6.1_±1.4_	11.4_±0.5_	8.2_±0.3_	7.9_±0.2_	11.7_±0.8_	x

**Table 6:** Timing and speedup for 4 × 4 ROIs with double precision.

Configurations	P100	vs	Xeon E5-2620	TITAN V	vs	Xeon E5-2620	units
	T1	T2	PD	T1	T2	PD	
CPU time	71.4_±1.4_	71.1_±2.0_	71.7_±1.9_	71.4_±1.4_	71.1_±2.0_	71.7_±1.9_	ms
GPU time	57.6_±16.2_	50.1_±9.9_	38.18_±0.2_	37.7_±2.2_	35.1_±2.4_	37.8_±2.5_	ms
GLRLM Speedup	1.2_±0.3_	1.4_±0.3_	1.9_±0.1_	1.9_±0.1_	2.0_±0.2_	1.9_±0.1_	x
CPU time	214.2_±3.7_	213.7_±5.5_	214.7_±6.1_	214.2_±3.7_	213.7_±5.5_	214.7_±6.1_	ms
GPU time	96.6_±27.6_	109.4_±27.9_	64.928_±0.9_	61.9_±3.0_	58.2_±4.8_	59.7_±4.0_	ms
0° feature Speedup	2.2_±0.7_	2_±0.7_	3.3_±0.1_	3.5_±0.2_	3.7_±0.3_	3.6_±0.2_	x
CPU time	214.9_±3.8_	214.9_±5.4_	215.8_±6.0_	214.9_±3.8_	214.9_±5.4_	215.8_±6.0_	ms
GPU time	35.2_±7.8_	46.8_±16.6_	23.9_±0.2_	29.3_±1.7_	27.2_±2.2_	26.8_±2.9_	ms
45° feature Speedup	6.1_±1.4_	4.6_±1.7_	9_±0.3_	7.4_±0.5_	7.9_±0.9_	8.1_±0.9_	x
CPU time	213.8_±3.6_	213.3_±5.6_	214.4_±6.1_	213.8_±3.6_	213.3_±5.6_	214.4_±6.1_	ms
GPU time	32.2_±11.1_	29.8_±8.4_	20.7_±0.3_	22.8_±1.1_	18.2_±1.4_	22.4_±1.9_	ms
90° feature Speedup	6.6_±2.3_	7.2_±2.1_	10.4_±0.4_	9.4_±0.5_	11.8_±1.1_	9.6_±0.9_	x
CPU time	213.9_±3.6_	213.6_±5.6_	214.7_±6.1_	213.9_±3.6_	213.6_±5.6_	214.7_±6.1_	ms
GPU time	36.1_±13.8_	37.9_±14.2_	23.8_±0.2_	24.4_±0.9_	23.1_±1.0_	23.6_±0.4_	ms
135° feature Speedup	5.9_±1.9_	5.6_±2.0_	9.0_±0.3_	8.8_±0.4_	9.3_±0.5_	8.8_±1.2_	x

## Discussion

There are some points observed from the results that can be elaborated. First, we noticed that for both CPU and GPU devices, performances are better with single precision, which is normal. Hence, our tests suggest that one should prefer the single precision mode in parallelization whenever the resulting precision is acceptable. However, on the other hand, the performances of single precision and double precision for GPUs do not differentiate themselves too much, which may seems paradoxical to the fact that theoretical peak performances of both Tesla P100 and Titan V GPUs are 2 times higher for single precision floating point arithmetics than double precision. A plausible explanation for the phenomena is that most of the work in our algorithm are done with integers, only a fraction of features computation involves floating point arithmetics. It can be observed from Tables 5 and 6, time for GLRLMs construction are almost the same, because only integer operations are employed. And for 7 out of 11 features as LRE, SRE, HGRE, etc., as long as there is no division in the numerator, the extraction can be done with almost all integer arithmetics except for the last division. Therefore, performance in single precision is only slightly better than in double precision.

Second, with the increase of ROI size, though the speedup drops, yet it remains much more stable than the method in the work^19^ of Tsai *et al*., as we don’t deal with low-level techniques directly and leaves them to the library CUB. In their work, a bunch of GLCM matrices must be built in parallel and features are then computed based on them. Our proposed paradigms would adapt to such similar tasks as claimed. The same logic can be applied to the GLCMs construction for each ROI, then features are computed with reduction by ROI index as long as there are some summation in the feature. Even better, we can combine the part of tailored matrix generation from the work of Tsai *et al*. and the feature extraction in our work, or vice versa. Brief, our method may suit other similar jobs that involve statistical computation.

However, there’s a shortcoming in such a general paradigm, the performance is not comparable to specific parallelization, as what Tsai *et al*. do in their work, which leads to the third point and the perspectives of future work, how to improve the performance of our algorithm. We find from Tables 5 and 6 that acceleration in GLRLMs construction is too weak. The reason is that duplication of overlapped ROIs and spreading them in global memory in the first step for constructing GLRLMs turns the original problem into a much too big one, which causes the parallel algorithm to deal with unnecessary work. In other words, the first rows concatenated for all ROIs actually overlaps a large part of the second rows concatenated from all ROIs, and similar things happens with third rows as well, and so on, which makes the following primitive reduce-by-key to do repeated reductions. Therefore an exchange of some steps in constructing GLRLMs may improve the performance. For example, we can scan each row of the image first, then we scatter the gray-level and run length pairs to an array, on which, we can do a segmented sort instead of a complete sort, etc. Nevertheless, these modifications are the cost we should pay for a less general paradigm.

## Conclusion

In this work, we propose a new paradigm that can be adapted to simultaneously deal with statistical computations for many overlapped ROIs on a single image by merely employing parallel primitives. We apply the idea to the task of constructing GLRLMs and extracting features in parallel for many ROIs of a MRI image as the example to illustrate the paradigm. Though, there is potential space to improve the acceleration reached by parallelization of our paradigm, experiments demonstrate that the paradigm remains to be a convenient and realistic way of implementing working prototypes for complicated problems with a reasonable performance boost.
