# Author Correction: GPU-Accelerated GLRLM Algorithm for Feature Extraction of MRI

**DOI:** 10.1038/s41598-020-67202-3

**Published:** 2020-06-23

**Authors:** Hanyu Zhang, Che-Lun Hung, Geyong Min, Jhih-Peng Guo, Meiyuan Liu, Xiaoye Hu

**Affiliations:** 1 Affiliated Cancer Hospital & Institute of Guangzhou Medical University, Guangzhou, China; 2 College of Computing and Informatics, Providence University, Taichung, Taiwan; 3 Department and Graduate Institute of Computer Science and Information Engineering, Chang Gung University, Tao-Yuan City, Taiwan; 4 Division of Rheumatology, Allergy and Immunology, Chang Gung Memorial Hospital, Taoyuan City, Taiwan; 5 AI Innovation Research Center, Chang Gung University, Taoyuan City, Taiwan; 6 Department of Computer Science and Communication Engineering Providence University, Taichung, Taiwan; 7 Laboratoire Mathématiques et Informatique pour la Complexité et les Systèmes, CentraleSupélec, 91190 Gif-sur-Yvette, France; 8 Department of Mathematics and Computer Science, University of Exeter, North Park Road, Exeter, EX4 4QF UK

Correction to: *Scientific Reports* 10.1038/s41598-019-46622-w, published online 26 July 2019

In the original version of this Article, Hanyu Zhang and Che-Lun Hung were incorrectly affiliated with ‘Affiliated Cancer Hospital & Institute of Guangzhou Medical University, Guangzhou, China’. The correct affiliations are listed below.

Hanyu Zhang:

College of Computing and Informatics, Providence University, Taichung, Taiwan.

Laboratoire Mathématiques et Informatique pour la Complexité et les Systèmes, CentraleSupélec, 91190, Gif-sur-Yvette, France.

Che-Lun Hung:

Department and Graduate Institute of Computer Science and Information Engineering, Chang Gung University, Tao-Yuan City, Taiwan.

Division of Rheumatology, Allergy and Immunology, Chang Gung Memorial Hospital, Taoyuan City, Taiwan.

AI Innovation Research Center, Chang Gung University, Taoyuan City, Taiwan.

Department of Computer Science and Communication Engineering Providence University, Taichung, Taiwan.

This error has now been corrected in the PDF and HTML versions of the Article.

